# Visceral fat dysfunction is positively associated with hypogonadism in Chinese men

**DOI:** 10.1038/srep19844

**Published:** 2016-01-22

**Authors:** Ningjian Wang, Hualing Zhai, Bing Han, Qin Li, Yi Chen, Yingchao Chen, Fangzhen Xia, Dongping Lin, Yingli Lu

**Affiliations:** 1 Institute and Department of Endocrinology and Metabolism, Shanghai Ninth People’s Hospital, Shanghai Jiao Tong University School of Medicine, Shanghai, China

## Abstract

Visceral adiposity index (VAI) well mirrors visceral fat dysfunction. No study explored the association between low androgen and VAI. We aimed to determine whether VAI was associated with hypogonadism and sex hormones, and also whether it better predicted hypogonadism than other obesity indices. Our data were collected from 16 sites in East China. 2,759 men were enrolled. Hypogonadism was defined as total testosterone < 11.3 nmol/L. VAI was calculated in male: (waist circumference/(39.68 + (1.88 × BMI))) × (triglycerides/1.03) × (1.31/HDL). 484 (17.5%) hypogonadal men had significantly higher VAI. After adjusting for age, smoking, neck and hip circumference, diabetes and hypertension, VAI was inversely associated with total testosterone, estradiol and SHBG (*P* < 0.01). Higher quartiles of VAI were associated with significantly increasing odds of hypogonadism (*P* for trend < 0.01). The fully adjusted odds ratio was 5.88 (95 CI% 4.09, 8.46) for the highest quartile compared with the lowest quartile of VAI. Among all the indices investigated, VAI showed the largest area under the curve (*P* < 0.001). In conclusion, the VAI was significantly associated with a higher prevalence of hypogonadism in Chinese men. VAI also best predicted hypogonadism among obesity indices (waist, hip and neck circumference, BMI, waist-hip ratio and body adiposity index).

Hypogonadism in male has been well documented to be a risk factor for diabetes^1^ and cardiovascular disease^2^. It is also common, especially in the middle aged and elderly. In USA, the prevalence of hypogonadism was 38.7% in men aged ≥45 years^3^ and in Chinese men, about a quarter had androgen deficiency^4^. The number is much higher among patients referring for the treatment of obesity, although the prevalence of symptomatic hypogonadism in the general population is very low, about only 2–3%^5^.

Hypogonadism has a bidirectional relationship with obesity^5^ and it has a strongest association with visceral obesity among the metabolic syndrome components^6^. The most common index to define visceral obesity is waist circumference (WC). However, another index called visceral adiposity index (VAI) shows promising capability to be a marker of visceral fat dysfunction, which has been reported in more than 70 publications since it was first introduced in 2010^7^,^8^. The VAI has been investigated in cardiovascular and cerebrovascular diseases^8^, nonalcoholic fatty liver disease^9^, polycystic ovary syndrome^10^, and acromegaly^11^. A recent study suggests, among the most common indices of adiposity including body mass index (BMI), WC, hip circumference and waist-hip ratio (WHR) and several adipocytokines, the VAI would be a simple tool for clearly reflecting adipose tissue dysfunction and associated cardiometabolic risk^12^. Amato *et al*. suggested VAI be applied in patients with hypogonadism^7^, but until now there is no study exploring the association between hypogonadism and VAI.

We performed a large investigation in 2014, referred to as **S**urvey on **P**revalence in **E**ast **C**hina for Me**t**abolic Diseases and Risk Factors (SPECT-China). Using the data from the study, we aimed to determine whether the VAI was associated with hypogonadism and sex hormones, and also whether VAI better predicted hypogonadism than other obesity indices. To the best of our knowledge, the current analyses are the first to explore the application of VAI in male hypogonadism.

## Results

### Characteristics of the study population

General characteristics of the study population are summarized in Table 1. Four hundred and eighty-four (17.5%) men were diagnosed with hypogonadism by total testosterone (T). Compared to men without hypogonadism, the men with hypogonadism had comparable age but had significantly higher VAI, waist, neck and hip circumference, BMI, other obesity indices and triglycerides (TG) as well as higher prevalence of diabetes, hypertension and metabolic syndrome. These men also had significantly lower estradiol (E2), sex hormone binding globulin (SHBG) and luteinizing hormone (LH).

**Figure 1:**
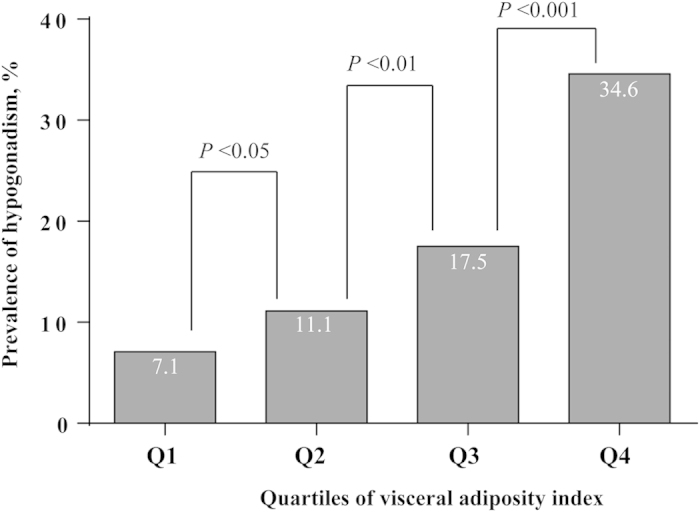
The prevalence of hypogonadism by quartiles of visceral adiposity index. *P* values were age-adjusted.

Table 2 shows the results of variables according to the VAI quartiles. The quartile ranges were ≤0.85, 0.86–1.32, 1.33–2.17 and ≥2.18. According to trend analysis, with the increase of VAI quartiles, total T, E2, SHBG, follicle-stimulating hormone (FSH) and LH decreased (all *P* for trend <0.001) and as expected, waist, neck and hip circumference, BMI, other obesity indices, TG, low density lipoprotein (LDL) as well as prevalence of hypertension and diabetes increased (all *P* for trend <0.001). We also observed the prevalence of hypogonadism of each VAI quartile was significantly higher than that of previous quartile (Fig. 1) (all age-adjusted *P* < 0.05).

### Association of VAI with sex hormones

Table 3 summarizes the results of the linear regression models studying the association of VAI with total T, E2, SHBG, LH and FSH. In the model adjusted for age and smoking, the VAI was inversely associated with total T (*Beta* = −0.255), E2 (*Beta* = −0.063), log-SHBG (*Beta* = −0.226) and log-FSH (*Beta* = −0.038) (all *P* < 0.05). After additional adjustments for neck and hip circumference, the association of VAI with total T, E2 and log-SHBG weakened but it was still significant (all *P* < 0.01). After additional adjustments for diabetes and hypertension, for total T (*Beta* = −0.202), E2 (*Beta* = −0.052) and log-SHBG (*Beta* = −0.159), the association with VAI did not change (all *P* < 0.01).

### Association of VAI with hypogonadism

Table 4 demonstrates the results of the logistic regression measuring the association of VAI with hypogonadism. In each model, the odds ratio (OR) for hypogonadism increased across VAI quartiles (all *P* for trend < 0.001). In the model adjusted for age and smoking (Table 4, model 1), compared with men in the lowest quartile of VAI, the OR of hypogonadism in men in the highest quartile of VAI were 7.76 (95% CI 5.49, 10.96; *P* < 0.001). After additional adjustments for obesity indices (neck and hip circumference), OR decreased and the association weakened (Table 4, model 2) but it was still significant (*P* for trend < 0.001). Additional adjustment for diabetes and hypertension also did not change this association (Table 4, model 3). The OR of hypogonadism in Q4 was 5.88 (95% CI 4.09, 8.46; *P* < 0.001). No interaction was observed between VAI, and smoking, neck circumference, hip circumference, diabetes and hypertension.

### AUCs of different obesity indices to predict hypogonadism

We explored the value of obesity indices in distinguishing between subjects with and without hypogonadism (Table 5). Using ROC analysis, VAI could distinguish subjects with hypogonadism from those without, with a sensitivity of 0.68, a specificity of 0.63. Its AUC was 0.71 (95% CI 0.69, 0.73; *P* < 0.001). WC, BMI, neck circumference, hip circumference, waist-hip ratio (WHR) and body adiposity index (BAI) was significantly inferior to VAI model in AUC (all *P* < 0.001).

### Sensitivity analyses

After excluding subjects with metabolic syndrome based on the International Diabetes criteria definition (n = 463), the OR for hypogonadism still increased across VAI quartiles in each model (all *P* for trend < 0.001). In fully adjusted model, the OR of hypogonadism in men in the highest quartile of VAI were 6.63 (95% CI 4.46, 9.86; *P* < 0.001). Its AUC was 0.70 (95% CI 0.66, 0.73; *P* < 0.001), still the largest among the obesity indices (all *P* < 0.01).

## Discussion

**Figure 2:**
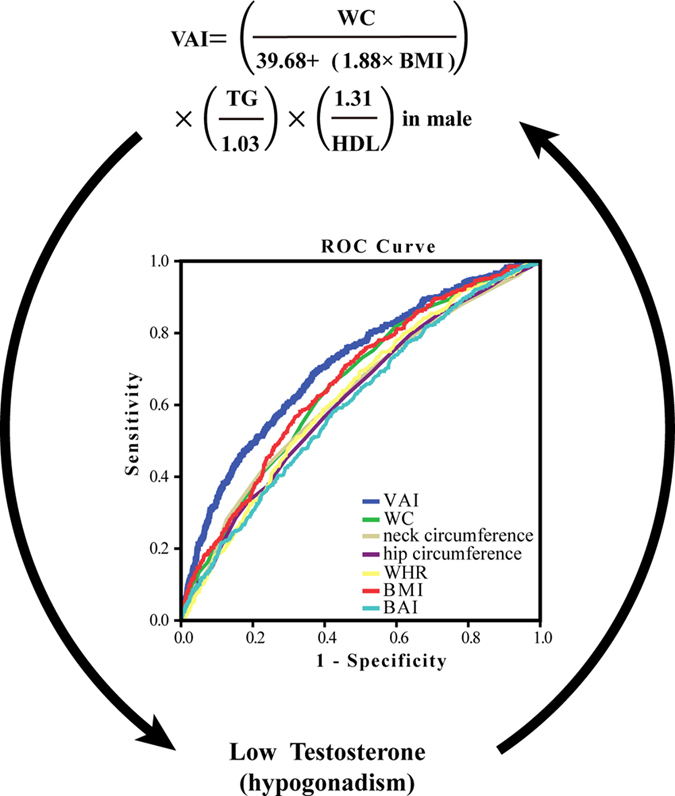
Relationship between VAI and hypogonadism in male. We observed that the VAI was significantly associated with lower total testosterone and hypogonadism. VAI also had the largest area under curves among different obesity indices, indication it best predicted hypogonadism. VAI, visceral adiposity index; WC, waist circumference; WHR, waist-hip ratio; BMI, body mass index; BAI, body adiposity index. This figure was created by Adobe Illustrator CS5 (Adobe Systems Incorporated, USA).

In this population-based study, we observed that the VAI mirroring visceral adipose tissue dysfunction was significantly associated with lower total T, E2 and SHBG levels after adjusting for age, smoking, neck and hip circumference, diabetes and hypertension. The fully adjusted OR of hypogonadism increased by 588% for the highest quartile compared with the lowest quartile of VAI. VAI also best predicted hypogonadism among different obesity indices. To the best of our knowledge, this study is the first to explore the association between VAI and hypogonadism (Fig. 2).

The VAI is a new method for assessment of adipose distribution and function, first presented by Marco Calogero Amato *et al*. in 2010^8^. It is a gender-specific empirical-mathematical model. VAI is determined by anthropometric (BMI and WC) and corrected for functional parameters (TG and HDL), which shows a strong relationship with visceral fat mass detected by MRI^8^. In a previous study, VAI was independently associated with both cardiovascular and cerebrovascular events, all metabolic syndrome factors, insulin resistance^8^ and adiponectin^13^. However, this was not observed for WC^8^. This further indicates WC alone could not well distinguish subcutaneous and visceral fat mass^14^. Given the association between hypogonadism and obesity, in our study we also found the VAI had significantly larger AUC than other common obesity indices including WC, BMI, neck circumference, hip circumference, WHR and BAI. Thus VAI may be used as a surrogate of visceral fat dysfunction and we added new evidence to the application of VAI.

Obesity and male hypogonadism are often associated. This is consistent with our findings that further indicates visceral fat mass or dysfunction may be associated with hypogonadism. How obesity induces hypogonadism is not completely clear. Hypothalamic dysfunction with a decreased gonadotropin-releasing hormone release seems to drive part or most of the mechanisms explaining hypogonadism in obese subjects^5^,^15^. From our data, we could also speculate that the hypothalamic-pituitary level is the probable origin of the hypogonadal condition, because in the hypogonadal group, E2, FSH and LH were lower, which was more evident when quartiles of VAI were considered. In clinical intervention studies, a meta-analysis reported bariatric surgery-induced weight loss which was dramatically more effective than life-style changes, induced a significant increase in T levels, SHBG, FSH and LH^16^. Hypogonadism may also induce visceral obesity. In surgically castrated animal model, weight significantly increased, especially along with a 7-fold increase in visceral fat accumulation in 2 months^17^, which is consistent with the phenomenon in men with androgen deprivation therapy by surgical or medical castration^18^. Moreover, testosterone replacement alters the cell size in visceral fat but not in subcutaneous fat in hypogonadal aged male rats^19^. It also prevents gain in visceral adipose tissue in aging men^20^. Therefore, visceral fat dysfunction, which could be reflected by VAI rather than by WC or BMI, may form a vicious circle with hypogonadism. A combined approach of lifestyle intervention (weight loss) and testosterone supplementation might be adopted as a further option to break this vicious circle in symptomatic, hypogonadal, obese men^5^.

Another interesting finding is not only T but also SHBG was inversely associated with VAI. Currently plasma SHBG could be recognized as a biomarker of the degree of inflammation in metabolic disorders^21^. Cytokines such as tumor necrosis factor alpha, interleukin 1 beta and adiponectin, all of which could be released by adipose tissue, regulate SHBG expression^21^. This further indicates the effect of visceral fat dysfunction on the sex hormones.

Our study had several strengths. First, regarding the novelty, this study is the first to detect an association between VAI and hypogonadism in general Chinese men, a largest population group in the world, thereby adding new evidence in this field. Second, because the anthropometric measurements and questionnaires were all completed by the same trained group and all the biomedical measurements were performed in one laboratory certified by the College of American Pathologists, this study has strong quality control. Third, because the SPECT-China study was performed in a general population, our results are more reflective as opposed to a clinic-based population. However, our study has several limitations. First, due to the cross-sectional nature of the study, we cannot draw a causal relationship between VAI and hypogonadism, however we have mentioned their association might be bidirectional. Second, this study recruited primarily Han Chinese men but VAI was set up based on the Caucasian population. However, the application of VAI in the Chinese population has been reported in about 10 studies and VAI is well associated with cardiovascular diseases^22^, fatty liver^23^, diabetes^24^ and insulin resistance^25^ in Chinese. Finally, we did not measure albumin, so the calculated free testosterone could not be obtained, which is a reliable index of free testosterone^26^. However, the most widely accepted parameters to establish the presence of hypogonadism is serum total testosterone^27^, which has been used by numerous studies^28^,^29^.

In conclusion, the VAI was significantly associated with a higher prevalence of hypogonadism in Chinese men. VAI also best predicted hypogonadism among common obesity indices. This study may have particular interest for some Asian populations including Chinese. They are more prone to have generally low BMI but visceral fat accumulation^30^,^31^, so hypogonadism may be further neglected and the application of VAI may improve this. Future studies should further explore the underlying mechanisms.

## Methods

### Study population

SPECT-China is a population-based cross-sectional survey on the prevalence of metabolic diseases and risk factors in East China (ChiCTR-ECS-14005052, www.chictr.org) from February to June 2014. A stratified cluster sampling method was used. This study was performed in Shanghai, Jiangxi Province and Zhejiang Province (three sites in urban areas of Shanghai, one site in an urban area of Jiangxi Province, three sites in rural areas in Shanghai, three sites in rural areas in Zhejiang and six sites in rural areas in Jiangxi Province), with detailed information in previous published articles^32^. We included Chinese citizens aged 18 years and older and excluded those who had severe communication problems or acute illnesses or were unwilling to participate. The study protocol was approved by the Ethics Committee of Shanghai Ninth People’s Hospital, Shanghai JiaoTong University School of Medicine. All procedures followed were in accordance with the ethical standards of the responsible committee on human experimentation (institutional and national) and with the Helsinki Declaration of 1975, as revised in 2008. All participants provided written informed consent before data collection.

A total of 6,899 subjects were enrolled in the SPECT-China study^32^. Among them, there were 2940 men who did not receive testosterone supplementation. Men with missing values of BMI or WC (n = 173), lipid profile (n = 2) and total testosterone (n = 6) were excluded from the study. Finally, this study included a total number of 2759 men with a mean (SD) age of 53 (13) years.

### Measurements

In every site, the same trained staff group completed the questionnaire including information on demographic characteristics, medical history and lifestyle risk factors, and collected anthropometric data. Current smoking was defined as having smoked at least 100 cigarettes in one’s lifetime and currently smoking cigarettes^33^. Body weight, height, and blood pressure were measured using standard methods, as previously described^33^. BMI was calculated as weight in kilograms divided by height in meters squared. WC was measured midway between the inferior margin of the last rib and the crest of the ilium in a horizontal plane^12^. Hip circumference was measured around the pelvis at the point of maximal protrusion of the buttocks^12^. WHR was calculated by dividing the WC by the hip circumference. Neck circumference was measured. Participants stood erect with their head positioned in the Frankfort horizontal plane. The superior border of a tape measure was placed just below the laryngeal prominence and applied perpendicular to the long axis of the neck^34^. BAI was calculated as hip circumference (in cm) divided by height (in m)^1.5^ −18^35^.

The participants fasted for 8 h before the study. Fasting blood samples were drawn between 0700 h and 1000 h. The blood samples for the plasma glucose test were collected in vacuum tubes with anticoagulant sodium fluoride and centrifuged on the spot within 1 h after collection. The blood samples were stored at −20 °C and were shipped in dry ice within 2 to 4 hours of collection to a central laboratory. Hemoglobin A1c was assessed using high-performance liquid chromatography (Medconn, MQ-2000PT, Shanghai, China). Plasma glucose and lipid profile including TG, high density lipoprotein (HDL) and LDL were measured by BECKMAN COULTER AU 680 (Germany). The total T, E2, FSH and LH were measured using the immulite 2000 platform chemiluminescence immunoassays (Siemens, Germany) and sex hormone binding globulin (SHBG) by electrochemiluminescence (Roche Cobas E601, Switzerland). The minimal detectable limit for hormones was as follows: 0.7 nmol/L (total T), 73.4 pmol/L (E2), and 0.1 IU/L (FSH and LH). The inter-assay coefficients of variation were as follows: 6.6% (total T), 7.5% (E2), 7% (SHBG), 4.5% (FSH) and 6.0% (LH). The intra-assay coefficients of variation were as follows: 5.7% (total T), 7% (SHBG), 6.2% (E2), 3.8% (FSH) and 4.9% (LH).

### Definition of variables and outcomes

Hypogonadism was defined as total T < 11.3 nmol/L in men^29^. The VAI was calculated according to the following equation^7^ where WC was expressed in centimeter, and TG and HDL levels in mmol/L: (WC/(39.68 + (1.88 × BMI))) × (TG/1.03) × (1.31/HDL). Based on the American Diabetes Association 2014 criteria, diabetes was defined as a previous diagnosis by healthcare professionals, fasting plasma glucose ≥7.0 mmol/L, or HbA1c ≥6.5%. Hypertension was defined as a systolic blood pressure of 140 mm Hg or higher or a diastolic blood pressure of 90 mm Hg or higher or current use of antihypertensive treatment. Metabolic syndrome was defined based on the International Diabetes Federation criteria^36^.

### Statistical analysis

We performed survey analyses using IBM SPSS Statistics, Version 22 (IBM Corporation, Armonk, NY, USA). All of the analyses were two-sided. *P* < 0.05 was considered to be statistically significant. The general characteristics are summarized as the mean (SD) values for continuous variables or as a number with proportion for categorical variables. To test for differences in characteristics between the participants with and without hypogonadism, Mann-Whitney U or Student T test was used for continuous variables, and the Pearson χ^2^ test was used for dichotomous variables. The values of total T (n = 1) and E2 (n = 754) under the minimal detectable limit were given a value midway between zero and the minimal detectable limit for the analyses: 0.35 nmol/L for total T and 36.7 pmol/L for E2.

The association of VAI (independent variable) with total T, E2, SHBG, FSH and LH (dependent variables) was assessed using linear regression. Model 1 controls for age (continuous variable) and smoking. Model 2 additionally controls for neck and hip circumference (both continuous variables). Because BMI and waist circumference were not adjusted because they are included in the equation of VAI. Model 3 additionally controls for diabetes and hypertension. SHBG, LH and FSH were log-transformed because of their skewed distribution. The results were expressed as standardized coefficients (beta) and standard errors.

The VAI was divided into quartiles. The first quartile represented the lowest one and the fourth quartile represented the highest. Odds ratio (OR) and 95% CI were calculated using logistic regression to determine the risk of hypogonadism for each quartile of VAI, with the lowest quartile as the reference. Models were the same as those in linear regression. We also tested the interaction effect between VAI and smoking, neck and hip circumference, diabetes and hypertension by adding a multiplicative factor in the logistic regression model.

To measure the accuracy of obesity indices in distinguishing between subjects with and without hypogonadism, we calculated the area under the curve (AUC) of the receiver operating characteristic (ROC) using standard methods^37^. Z test was performed to examine differences between AUCs.

Marco Calogero Amato *et al*. suggests VAI better be used in subjects without overt metabolic syndrome^7^. In sensitivity analyses, we excluded subjects with metabolic syndrome (n = 463) and then reperformed the logistic regression and calculate AUCs.

## Additional Information

**How to cite this article**: Wang, N. *et al*. Visceral fat dysfunction is positively associated with hypogonadism in Chinese men. *Sci. Rep*. **6**, 19844; doi: 10.1038/srep19844 (2016).

## Figures and Tables

**Table 1:** General characteristics of the participants.

	Men without hypogonadism	Men with hypogonadism	*P*
*N*	2275	484	
Age, yr	53 (14)	52 (12)	0.78
Metabolic factors			
Body mass index, kg/m^2^	24.2 (3.2)	26.1 (3.5)	<0.001
Waist circumference, cm	82 (9)	87 (9)	<0.001
Neck circumference, cm	35.6 (3.1)	36.8 (2.8)	<0.001
Hip circumference, cm	93 (6)	96 (7)	<0.001
Waist-hip ratio	0.88 (0.17)	0.93 (0.46)	<0.001
Body adiposity index, %	25.0 (3.3)	26.3 (3.6)	<0.001
Triglycerides, mmol/L	1.69 (1.46)	2.96 (3.26)	<0.001
HDL, mmol/L	1.39 (0.31)	1.27 (0.30)	<0.001
LDL, mmol/L	2.92 (0.70)	2.98 (0.67)	0.08
Visceral adiposity index	1.68 (2.00)	3.26 (4.43)	<0.001
Hypertension, %	39.9	49.5	<0.001
Diabetes, %	10.8	23.1	<0.001
Metabolic syndrome, n (%)	315 (13.8)	148 (30.6)	<0.001
Sex hormones			
Total T, nmol/L	17.6 (5.2)	9.2 (1.9)	<0.001
E2, pmol/L	112.2 (65.5)	83.3 (53.5)	<0.001
SHBG, nmol/L	49.0 (24.9)	29.4 (16.4)	<0.001
FSH, IU/L	8.7 (7.4)	9.6 (10.8)	0.66
LH, IU/L	5.5 (3.7)	5.1 (4.6)	<0.001
Current smoker, %	49.2	43.3	<0.05

E2, estradiol; FSH, follicle-stimulating hormone; LH, luteinizing hormone; SHBG, sex hormone binding globulin; T, testosterone.

The data are summarized as the mean (standard deviation) for continuous variables, or as number with proportion for categorical variables. The Mann-Whitney U or Student T test was used for continuous variables, and the Pearson χ^2^ test was used for dichotomous variables.

**Table 2:** Characteristics of the participants by quartiles of visceral adiposity index.

Visceral adiposity index	Q1	Q2	Q3	Q4	*P for trend*
	≤0.85	0.86–1.32	1.33–2.17	≥2.18	
*N*	693	687	691	688	
Age, yr	54 (15)	53 (14)	52 (13)	51 (12)	<0.001
Metabolic factors					
Body mass index, kg/m^2^	22.6 (3.0)	24.0 (3.1)	25.2 (3.1)	26.2 (3.0)	<0.001
Waist circumference, cm	77 (8)	81 (9)	85 (8)	88 (8)	<0.001
Neck circumference, cm	34.4 (2.4)	35.4 (2.6)	36.4 (3.8)	37.0 (2.6)	<0.001
Hip circumference, cm	91 (6)	93 (6)	95 (6)	96 (6)	<0.001
Waist-hip ratio	0.85 (0.07)	0.90 (0.48)	0.89 (0.07)	0.92 (0.06)	<0.001
Body adiposity index, %	24.1 (3.4)	24.9 (3.4)	25.7 (3.4)	26.1 (3.2)	<0.001
Triglycerides, mmol/L	0.84 (0.19)	1.24 (0.26)	1.75 (0.38)	3.84 (3.15)	<0.001
HDL, mmol/L	1.64 (0.31)	1.41 (0.25)	1.29 (0.23)	1.14 (0.23)	<0.001
LDL, mmol/L	2.69 (0.65)	2.92 (0.73)	3.05 (0.67)	3.05 (0.67)	<0.001
Hypertension, %	35.9	40.7	40.4	49.4	<0.001
Diabetes, %	10.5	10.0	11.0	20.3	<0.001
Sex hormones					
Total T, nmol/L	19.0 (6.4)	16.8 (5.4)	15.4 (5.2)	13.3 (4.3)	<0.001
E2, pmol/L	119.3 (73.0)	107.2 (60.2)	103.7 (60.9)	98.0 (61.1)	<0.001
SHBG, nmol/L	59.7 (29.2)	47.9 (22.1)	40.8 (21.5)	33.6 (16.8)	<0.001
FSH, IU/L	10.4 (11.1)	8.8 (7.2)	8.6 (6.3)	7.5 (6.5)	<0.001
LH, IU/L	6.3 (5.3)	5.4 (3.5)	5.1 (3.1)	4.7 (3.1)	<0.001
Current smoker, %	44.2	43.2	51.6	53.8	<0.001

E2, estradiol; FSH, follicle-stimulating hormone; LH, luteinizing hormone; SHBG, sex hormone binding globulin; T, testosterone. The data are summarized as the mean (standard deviation) for continuous variables, or as number with proportion for categorical variables. *P* for trend was calculated by ANOVA and Chi-square test.

**Table 3:** Association of visceral adiposity index with sex-related hormones.

Dependent variables	Visceral adiposity index (independent variable)		
	Model 1	Model 2	Model 3
Total T	−0.255 (0.042)^‡^	−0.212 (0.042)^‡^	−0.202 (0.042)^‡^
E2	−0.063 (0.483)^†^	−0.054 (0.492)^†^	−0.052 (0.495)^†^
log-SHBG	−0.226 (0.001)^‡^	−0.170 (0.001)^‡^	−0.159 (0.001)^‡^
log-LH	−0.031 (0.002)	−0.008 (0.002)	−0.009 (0.002)
log-FSH	−0.038 (0.002)^*^	−0.029 (0.002)	−0.032 (0.002)

Beta coefficients (standard errors) from linear regression models are presented. *P < 0.05, ^†^*P* < 0.01, ^‡^*P* < 0.001.

T, testosterone; E2, estradiol; SHBG, sex hormone binding globulin.

Model 1 controls for age (continuous variable) and smoking. Model 2 additionally controls for neck and hip circumference (both continuous variables). Model 3 additionally controls for diabetes and hypertension. SHBG, LH and FSH were log-transformed because of their skewed distribution.

**Table 4:** Association of visceral adiposity index with hypogonadism.

Visceral adiposity index	Model 1	Model 2	Model 3
Q1 (≤0.85)	1.00 (ref.)	1.00 (ref.)	1.00 (ref.)
Q2 (0.86–1.32)	1.71 (1.16, 2.52)	1.61 (1.09, 2.39)	1.62 (1.09, 2.41)
Q3 (1.33–2.17)	3.06 (2.13, 4.41)	2.64 (1.81, 3.84)	2.63 (1.81, 3.84)
Q4 (≥2.18)	7.76 (5.49, 10.96)	6.33 (4.41, 9.08)	5.88 (4.09, 8.46)
*P* value for trend	<0.001	<0.001	<0.001

The data are expressed as odds ratio (95% CI) unless otherwise indicated. Logistic regression analysis was used.

Model 1 controls for age (continuous variable) and smoking. Model 2 additionally controls for neck and hip circumference (both continuous variables). Model 3 additionally controls for diabetes and hypertension.

**Table 5:** AUC for visceral adiposity index and other adiposity indices for hypogonadism.

Prediction model	AUC ROC	SE for AUC	*P* value 1	*P* value 2
Visceral adiposity index	0.71 (0.69, 0.73)	0.01	<0.001	
Waist circumference	0.66 (0.64, 0.67)	0.01	<0.001	<0.001
Neck circumference	0.63 (0.61, 0.65)	0.01	<0.001	<0.001
Hip circumference	0.62 (0.60, 0.64)	0.01	<0.001	<0.001
Waist-hip ratio	0.63 (0.61, 0.64)	0.01	<0.001	<0.001
Body mass index	0.66 (0.65, 0.68)	0.01	<0.001	<0.01
Body adiposity index	0.60 (0.59, 0.62)	0.01	<0.001	<0.001

AUC, area under the curve; ROC, receiver operating characteristic.

*P* value 1: The diagnostic value for ROC, two tail significance.

*P* value 2: Difference of AUC compared to the visceral adiposity index model, two tail significance (Z test).
